# High-risk spatial clusters for Zika, dengue, and chikungunya in Rio de Janeiro, Brazil

**DOI:** 10.11606/s1518-8787.2023057004932

**Published:** 2023-05-30

**Authors:** Reinaldo Souza-Santos, Andrea Sobral, Andre Reynaldo Santos Périssé

**Affiliations:** I Fundação Oswaldo Cruz Escola Nacional de Saúde Pública Sergio Arouca Departamento de Endemias Samuel Pessoa Rio de Janeiro RJ Brasil Fundação Oswaldo Cruz. Escola Nacional de Saúde Pública Sergio Arouca. Departamento de Endemias Samuel Pessoa. Rio de Janeiro, RJ, Brasil

**Keywords:** Zika, Dengue, Chikungunya, Epidemiology, Spatial Analysis, Cluster Detection, Ecological Studies

## Abstract

**OBJECTIVE:**

To analyze the spatial distribution and identify high-risk spatial clusters of Zika, dengue, and chikungunya (ZDC), in the city of Rio de Janeiro, Brazil, and their socioeconomic status.

**METHODS:**

An ecological study based on data from a seroprevalence survey. Using a rapid diagnostic test to detect the arboviruses, 2,114 individuals were tested in 2018. The spatial distribution was analyzed using kernel estimation. To detect high-risk spatial clusters of arboviruses, we used multivariate scan statistics. The Social Development Index (SDI) was considered in the analysis of socioeconomic status.

**RESULTS:**

Among the 2,114 individuals, 1,714 (81.1%) were positive for at least one arbovirus investigated. The kernel estimation showed positive individuals for at least one arbovirus in all regions of the city, with hot spots in the North, coincident with regions with very low or low SDI. The scan statistic detected three significant (p<0.05) high-risk spatial clusters for Zika, dengue, and chikungunya viruses. These clusters correspond to 35.7% (n=613) of all positive individuals of the sample. The most likely cluster was in the North (cluster 1) and overlapped regions with very low and low SDI. Clusters 2 and 3 were in the West and overlapping regions with low and very low SDI, respectively. The highest values of relative risks were in cluster 1 for CHIKV (1.97), in cluster 2 for ZIKV (1.58), and in cluster 3 for CHIKV (1.44). Regarding outcomes in the clusters, the Flavivirus had the highest frequency in clusters 1, 2, and 3 (42.83%, 54.46%, and 52.08%, respectively).

**CONCLUSION:**

We found an over-risk for arboviruses in areas with the worst socioeconomic conditions in Rio de Janeiro. Moreover, the highest concentration of people negative for arboviruses occurred in areas considered to have better living conditions.

## INTRODUCTION

The co-circulation of dengue, Zika, and chikungunya viruses has been reported in different studies from different countries, but mainly in Brazil. Most were developed based on secondary data from health services^1^, and few were based on territorial-based seroprevalence primary data^6^.

The relevance of epidemiological studies on these arboviruses has been widely presented. Besides, they indicate the need for better knowledge about the prevalence in different population groups and identifying areas with higher risk, socioeconomic, demographic, and environmental characteristics of these areas^7^.

According to Power et al.^8^, low socioeconomic status can increase the risk of arbovirus infection. The authors reported that due to the non-standardization of measures of poverty, income, and social vulnerability among the studies from different countries included in the sample, these variables were not included in the meta-analysis. On the other hand, descriptive analyzes of the same studies and the literature on social determinants of health indicate lower income as a risk factor. This scenario demonstrates the need for further studies to delineate the relationship between individual and community socioeconomic indicators and the risks of arbovirus infection to guide specific public health interventions.

Based on the seroprevalence survey of Zika, dengue, and chikungunya^6^, this study aimed to analyze the spatial distribution and identify high-risk spatial clusters of these arboviruses in the city of Rio de Janeiro, Brazil, and their socioeconomic status.

## METHODOS

### Study Design, Setting, and Participants

This is an analytical ecological study, having the city’s Administrative Region (AR) as the unit of analysis, and based on primary data from a cross-sectional seroprevalence study in Rio de Janeiro, Brazil, carried out by Périssé et al.^6^.

**Figure 1:**
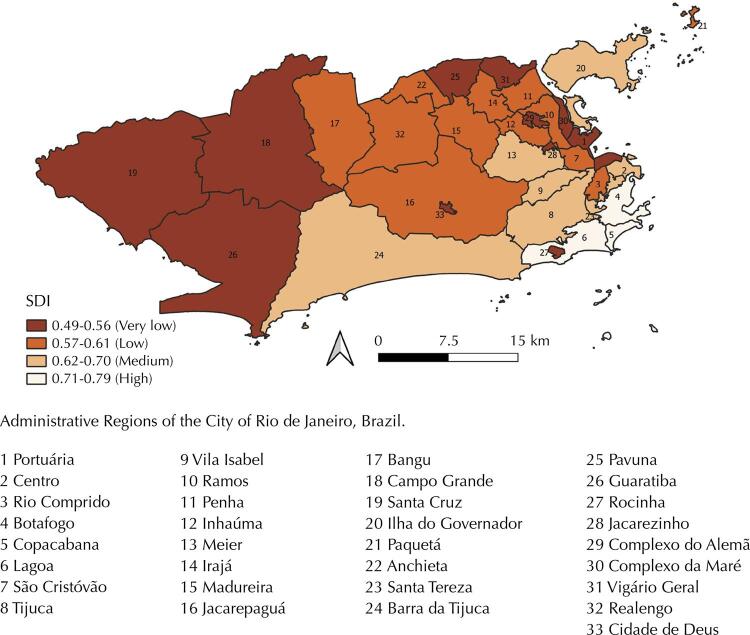
Social Development Index (SDI) and Administrative Regions of the City of Rio de Janeiro, Brazil.

Rio de Janeiro is located in the Southeastern macro-region of Brazil, and the Atlantic Ocean limits it to the south. The estimated population for 2018 was 6,688,927 (https://www.ibge.gov.br/). The climate in the city is tropical, hot, and humid, with local variations due to differences in altitude, vegetation, and proximity to the ocean (http://www.inpe.br/ accessed in February 2021). The average annual temperature between 1981 and 2010 was 29°C, with the highest daily temperature averages (from 30° to 32°C) occurring in the summer. Summertime is also the period with the greatest precipitations (an average of 205mm of precipitation in January between 1981 and 2010). The city is geographically organized into 33 ARs (Figure 1).

The 2,120 volunteers were tested with Rapid Diagnostic Test (RDT) for Zika, dengue, and chikungunya (ZDC) viruses between July and October 2018. Individuals with positive IgG and/or IgM results from each virus were classified as positive for each virus^6^.

According to Périssé et al.^6^, for the sample size calculation, the three arboviruses’ prevalence was considered with a minimum proportion of 1.5%, for which the relative margin of error of the estimate should be a maximum of 35%. Considering that the average number of residents per household was 2.93 in the city according to the 2010 census, the sample size of homes was 1,535. Thus, this was a representative sample of the entire population and regions of the city. During the application of RDT, the geographical coordinates of households were captured. The selection of participants was random, based on the distribution of homes by census sectors of the Brazilian Institute of Geography and Statistics (IBGE), ordered by the AR. Due to the lack of geographic coordinates, the analysis considered 2,114 individuals in 1,197 households with correctly registered coordinates.

The ARs shapefiles were generated from census tracks shapefiles obtained from IBGE (https://www.ibge.gov.br/geociencias/downloads-geociencias.html, accessed on January 05th 2020) using QGis 3.4.9 software.

### Variables

For the analysis of spatial distribution by kernel estimation, the set of RDT results for the three arboviruses were grouped into positive and negative. The RDT results for each arbovirus, positive, or negative, were used for cluster detection and characterization. The analysis of the spatial distribution of socioeconomic characteristics was based on the Social Development Index (SDI).

### Spatial Distribution

The kernel estimation was used to separately analyze the spatial distribution of individual positives and negatives for arboviruses by household geographic coordinates. Those that were positive for at least one of the viruses tested were considered positive for arbovirus. After testing parameters for the kernel analysis, the best fit was an 11 km radius and a grid with 200 columns, using the QGis 3.4.9 software. The digital map of ARs was superimposed on the kernel estimation results.

### Clusters Detection

To detect high-risk spatial clusters of ZDC in Rio de Janeiro, a multivariate Bernoulli scan statistic for multiple datasets was used, one for each arbovirus. This technique could simultaneously search for Zika, dengue, and chikungunya clusters using SaTScan™ software (v. 9.6).

Scan statistic was used to detect and evaluate purely spatial clusters. This procedure is done by gradually scanning a circular window across space and recording the number of observed and expected occurrences inside the window at each location. This technique calculates the log-likelihood ratio (LLR) for each cluster and the Relative Risk for each arbovirus. The LLR for a particular cluster is calculated by the sum of the LLR for the three arboviruses^9^.

For each location and size of the scanning window, the alternative hypothesis is that there is an elevated risk within the window compared to outside. The window with the maximum likelihood is the most likely cluster, that is, the cluster least likely to be due to chance. A p-value is assigned to this cluster.

For the Bernoulli model, the likelihood function is:


$${\left(\frac{c}{n}\right)}^{0}{\left(\frac{n-c}{n}\right)}^{n-o}{\left(\frac{C-c}{N-n}\right)}^{c-\theta }{\left(\frac{\left(N-n)-(C-c\right)}{N-n}\right)}^{\left(N-n)-(C-e\right)}I()$$

where *C* is the total number of cases, *c* is the observed number of cases within the window, *n* is the total number of cases and non-cases within the window, while *N* is the combined total number of cases and non-cases in the data set. *I( )* is an indicator function. When SaTScan is set to scan only for clusters with high rates, *I( )* is equal to 1.

The estimated risk within the cluster is divided by the estimated risk outside the cluster. The estimated risk is calculated as the observed cases divided by the expected cases within the cluster, which is then divided by the observed cases and divided by the expected cases outside the cluster. In mathematical notation, this is represented as:


$$RR=\frac{c/E[c]}{\left(C-c)/(E[C]-E[c]\right)}=\frac{c/E[c]}{\left(C-c)/(C-E[c]\right)}$$

where c is the number of observed cases within the cluster and C is the total number of cases in the data set. Since the analysis is conditioned on the total number of cases observed, *E[C] = C*.

Using a Bernoulli model, where a 0/1 variable represents cases and non-cases, and after testing several spatial parameters, we chose the maximum spatial cluster size of 24% of the population at risk and the maximum spatial cluster size of 8 km. The percentage of 24% was tested since it was the highest value of occurrence of one of the three arboviruses (dengue) in the sample population^6^. We considered statistically significant clusters (p < 0.05) with no geographical overlap.

Within each cluster, the frequency of RTD outcomes was analyzed. Individuals with positive results for both ZIKV and DENV were classified as flaviviruses (FLAV). The final RTD outcomes were ZIKV, DENV, CHIKV, ZIKV+CHIKV, DENV+CHIKV, Flavirirus, Flavivirus+CHIKV, and no arboviruses^6^.

RTD outcomes, inside and outside the clusters, were compared, and the chi-square test was used in the R software (v. 3.4.4). The digital map of ARs was superimposed on the clusters detection results.

### Socioeconomic Status

By analyzing the differences in socioeconomic status among ARs, a thematic map was elaborated with the Social Development Index (SDI). The SDI is a composite indicator that represents four main dimensions: housing conditions, sanitation, education, and income, based on eight indicators from the 2010 Brazilian Demographic Census. The SDI ranges from 0 to 1, with 0 accounting for places with the worst socioeconomic conditions and 1 for the best ones (10). The SDI was stratified in very low (0.49 – 0.56), low (0.57 – 0.61), medium (0.62 – 0.70), and high (0.71 – 0.76).

The indicators used to calculate the SDI were the following. Percentage of permanent private households with adequate water supply connected to the public distribution network. Percentage of permanent private households with adequate sewage connected to the public sewage or pluvial network. Percentage of permanent private households with garbage collected directly by cleaning service or placed in cleaning service bucket. Average number of bathrooms per resident (numerator = number of bathrooms in the permanent private household; denominator = total number of people in the permanent private household). Percentage of illiteracy among residents from 10 to 14 years old, concerning all residents in this age group. Per capita income of permanent private households, expressed in 2010 minimum wages. Percentage of private households with per capita household income up to one minimum wage. Percentage of private households with per capita household income exceeding five minimum wages^10^.

### Ethical Aspects

This study is under Resolution n. 466*/*2012, issued by the Brazilian National Ethics Research Committee and approved on April 06^th^, 2018 (CAAE 83186318.1.0000.5240).

## RESULTS

**Figure 2:**
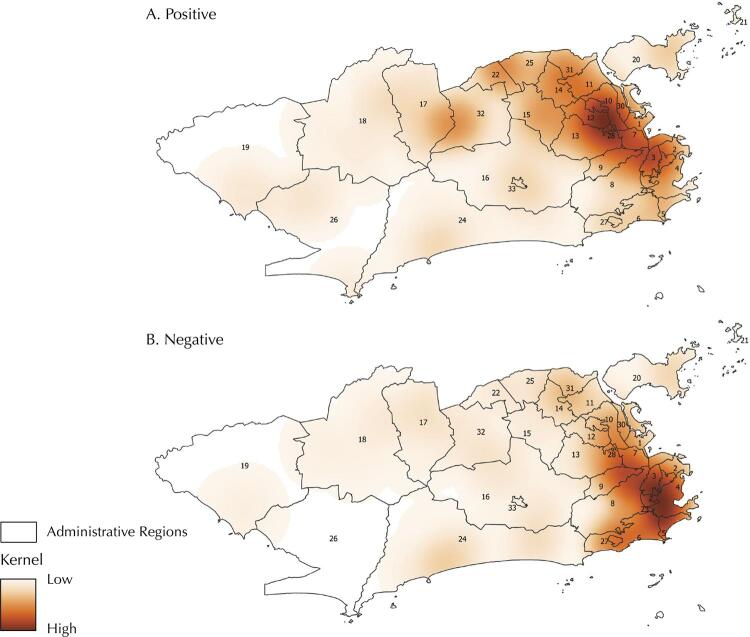
Kernel estimation of positive and negative individuals for Zika, dengue, and chikungunya, considering Administrative Regions of the City of Rio de Janeiro, Brazil, 2018.

Among 2,114 individuals, 1,714 (81.1%) were positive for at least one arbovirus investigated, and 400 (18.9%) were negative for the ZDC. The spatial distribution based on kernel estimation shows that positive individuals for at least one arbovirus are in all city territory, with hot spots in ARs in the North of the city that present very low or low SDI. Another hot spot with medium intensity in two ARs in the West with low SDI. Negative individuals for the investigated arboviruses were also observed across the city. However, with two hotspots of greater intensity. The first encompasses five ARs in the South, with high SDI, and the second encompasses four ARs in the North, with medium SDI. The overlap of the areas with greater intensity of positive and negative individuals is small and identified in three ARs in the north of the city with the greater intensity of negative individuals for ZDC (Figures 1 and 2).

**Figure 3:**
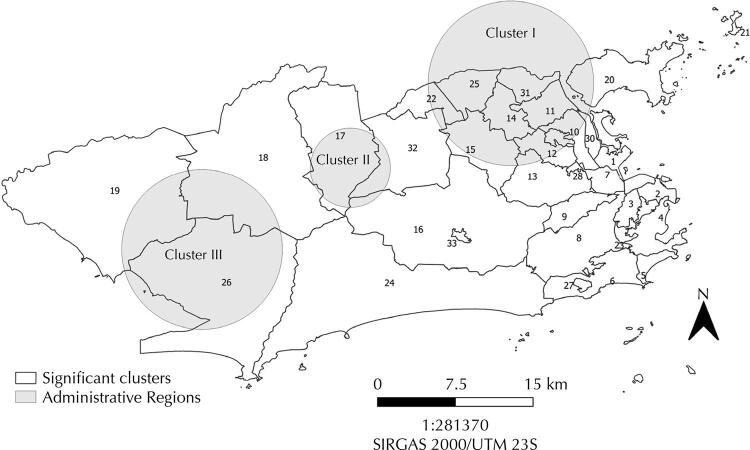
High-risk spatial clusters for Zika, dengue and chikungunya in Rio de Janeiro, Brazil, 2018.

The multiple datasets scan statistic identified three significant (p<0.05) high-risk spatial clusters for ZDC viruses. These clusters correspond to 35.7% (n=613) of all positives of the sample. The most likely cluster was in the North (cluster 1), overlapping nine ARs (LLR = 65.43) with very low and low SDI. Clusters 2 and 3 were located in the West. Cluster 2 overlaps two ARs (LLR = 28.27) with low SDI, and cluster 3 overlaps two ARs (LLR = 14.77) with very low SDI. Comparing RRs, the highest values were in cluster 1 for CHIKV (1.97), in cluster 2 for ZIKV (1.58), and in cluster 3 for CHIKV (1.44) (Table 1 and Figures 1 and 3).

**Table 1:** High-risk spatial clusters for seroprelavence of Zika, dengue, and chikungunya in Rio de Janeiro, Brazil, 2018.

Clusters							Total

	1		2		3		
Arboviruses	n	RR	n	RR	n	RR	n
DENV	429	1.19	103	1.2	47	1.31	579
ZIKV	315	1.34	90	1.58	33	1.4	440
CHIKV	140	1.97	27	1.36	12	1.44	179
p-value	< 0.05		< 0.05		< 0.05		
LLR	65.43		28.27		14.77		

**Administrative Regions**							

1	Penha		Bangu		Campo Grande		
2	Ramos		Realengo		Guaratiba		
3	Inhauma		Jacarepaguá^a^		Santa Cruz^a^		
4	Irajá		Campo Grande^a^				
5	Madureira						
6	Anchieta						
7	Pavuna						
8	Complexo do Alemão						
9	Vigário Geral						
10	Meier						
11	Ilha do Governador^a^						
12	Realengo^a^						

RR: relative risk; LLR: log-likelihood ratio.

^a^ ARs touched by the cluster boundary.

Regarding RTD outcomes in the clusters, the Flavivirus had the highest frequency in clusters 1, 2, and 3 (42.83%, 54.46%, and 52.08%, respectively). The frequency of Flavivirus+CHIKV was 23.18%, 18.75%, and 14.58%, respectively. For DENV alone they were 23.18%, 15.18%, and 22.92%, respectively. The CHIKV and ZIKV alone were not found in cluster 3 and had lower frequency in the other clusters. The ZIKV+CHIKV and DENV+CHIKV had lower frequencies in all clusters. The positive people for at least one arbovirus had high frequencies in clusters 1 and 2 (>90%). In cluster 3, 100% was positive. Comparing outcomes inside and outside the clusters, Flavivirus+CHIKV was more frequent inside the clusters (p<0.05). The frequencies for DENV, Flavivirus, and no arboviruses also had significant differences (p<0.05) (Table 2).

**Table 2:** Rapid diagnostic test outcomes of the sample of the population, considering spatial clusters in the city of Rio de Janeiro, Brazil, 2018.

	Cluster 1		Cluster 2		Cluster 3		All clusters		No cluster		Total	
Arboviruses	n	%	n	%	n	%	n	%	n	%	n	%
ZIKV	14	3.09	7	6.25	0	0.00	21	3.43	42	3.81	63	3.68
DENV	105	23.18	17	15.18	11	22.92	133	21.70	386	35.06^a^	519	30.28
CHIKV	8	1.77	1	0.89	0	0.00	9	1.47	31	2.82	40	2.33
ZIKV+CHIKV	2	0.44	1	0.89	1	2.08	4	0.65	12	1.09	16	0.93
DENV+CHIKV	25	5.52	4	3.57	4	8.33	33	5.38	50	4.54	83	4.84
Flavivirus	194	42.83	61	54.46	25	52.08	280	45.68	480	43.60^a^	760	44.34
Flavivirus+CHIKV	105	23.18	21	18.75	7	14.58	133	21.70	100	9.08^a^	233	13.59
Arbovirus	453	90.96	112	97.39	48	100.00	613	92.74	1.101	75.77	1.714	81.08
No arbovirus	45	9.04	3	2.61	0	0.00	48	7.26	352	24.23^a^	400	18.92
Total	498		115		48		661		1.453		2.114	

^a^ Clusters and no clusters χ^2^ = p < 0.05

## DISCUSSION

According to the literature, the spatial distribution of Zika, dengue, and chikungunya seroprevalence studies have been developed mainly based on secondary data^6,7^. Our results are the first based on the spatial analysis of a cross-sectional seroprevalence study, with a sample of individuals and their households in the area.

The distribution of arboviruses (Zika, dengue, and chikungunya) across the city in almost the entire sampled population (81.1%), with hot spots in worse living conditions areas. Other studies investigated seroprevalence for these three arboviruses separately but without considering their geographical distribution. In French Polynesia, the seroprevalence was >83% for dengue, 76% for chikungunya, and 22% for Zika^11^. In São José do Rio Preto, Brazil, the dengue seroprevalence was 74.6% in the sampled population^12^. According to Fritzell et al.^7^, there is heterogeneity for arboviruses seroprevalence between continents and within a given country for dengue, chikungunya, and Zika viruses, ranging from 0 to 100%, 76%, and 73%, respectively.

Several studies on dengue, Zika, or chikungunya geographical distribution, developed in different countries, have shown an association between low socioeconomic conditions and/or high population density and/or human mobility^8,13^. The results of the studies developed in Rio de Janeiro are similar to ours, pointing out areas with high population density and low socioeconomic status^1, 19^.

According to our results, the two hot spots of no arbovirus located in areas with better socioeconomic levels emphasize the relationship between living conditions and transmission of the diseases by *Aedes aegypti*. Moreover, Rio de Janeiro is a mosaic of areas with different socioeconomic conditions, coexisting with enormous *favelas* (Brazilian slums), with the worst socioeconomic levels, and areas with higher socioeconomic levels. The importance of spatial heterogeneity in the dengue epidemic, considering a local scale, was demonstrated by Favier et al.^22^. Furthermore, the house structure heterogeneity as a risk to virus transmission by *Ae. aegypti* was pointed out on a local scale study in California, USA^23^.

Multiple dataset scan analyses detected a primary cluster in the North and the other two in the city’s West zone, overlapping areas with low socioeconomic levels. The higher RRs were to CHIKV in clusters 1 (1.97) and 3 (1.44) and to ZIKV in cluster 2 (1.58). Freitas et al.^3^ found 16 clusters, of which nine showed dengue, chikungunya, and Zika coinciding in time and space. The primary cluster was predominantly located in the city’s Downtown region, and the other clusters are in the same areas detected in our study. In the primary cluster, the highest RRs were for dengue (21.16), chikungunya (25.30), and Zika (7.66). Freitas et al.^3^ used multivariate scan statistics for multiple datasets for this study, considering neighborhood centroids and secondary data from health services.

Santos et al.^24^ detected dengue clusters in the same areas of our study, overlapping North and West regions. The authors identified RRs similar to ours, ranging from 4.32 to 8.42 for a population younger than five years, and ranging from 2.69 to 7.93, depending on the year. To better fit into the territorial reality, the centroids’ locations were adjusted to the areas with the highest population density in each neighborhood.

Differences in the number of clusters and RR values are due to the kind of data and unit of analysis. Our study was based on the coordinates of households and a population sample. In Rio de Janeiro, the North and Central regions of the city have a more significant number of small neighborhoods and *favelas* with a higher population density. By using geographic coordinates of the residences and sample, respecting the distribution of the population by Administrative Region^6^, we obtained more reliable results. Furthermore, we used Bernoulli’s model, considering positives and negatives, other than the Poisson’s model, which was used by Freitas et al.^3^ and Santos et al.^24^.

Flavivirus RTD outcome had the highest frequency inside and outside the clusters. Flavivirus+CHIKV and DENV alone also had high frequency. We observed a similar scenario in a cross-sectional and observational study in Campo Grande, MS, Brazil. In 2016, 79.1% of the blood samples were for ZIKV and/or DENV infection and 5.6% for CHIKV^1^. On the other hand, the results of a study carried out in Fortaleza, CE, Brazil, based on secondary data, show a higher frequency of CHIKV infection. In 2016, 58.01% for CHIKV, and 41.99% for DENV/ZIKV infection. In 2017, 85.63% for CHIKV and 14.37% for DENV/ZIKV. The authors considered dengue cases indistinguishable from Zika cases from 2015 to 2017^4^.

Considering that the RTD use IgG and IgM simultaneously, our results do not allow an analysis of the simultaneous co-infection of these arboviruses during the RTD application. However, the population had contact with these viruses, making co-circulation evident in the city. Moreover, the highest frequency of non-arbovirus occurred outside the clusters, confirming the results of the kernel analysis and population socioeconomic levels and risk to arboviruses.

This study showed a scenario of over-risk for arboviruses in areas with the worst socioeconomic conditions in Rio de Janeiro. Furthermore, the highest concentration of negative people for arboviruses occurred in areas with better living conditions.

The results are based on data from a cross-sectional seroprevalence study with a representative sample of the entire population and regions of the city. Therefore, our findings reflect the ZDC’s actual occurrence and spatial distribution, which can support better surveillance and disease control actions.
